# Fear of Cancer Progression as a Psychological Component of Cancer Rehabilitation in Men Treated for Reproductive System Malignancies: A Multi-Regional Cross-Sectional Study in Kazakhstan

**DOI:** 10.3390/healthcare14182961

**Published:** 2026-09-10

**Authors:** Sayan Sagidullin, Gulmira Sagidullina, Khussan Umurzakov, Gulnar Shalgumbayeva, Marzhan Dauletyarova

**Affiliations:** 1 Center of Oncology and Surgery of the East Kazakhstan Region, Oskemen 070000, Kazakhstan; satasata@inbox.ru; 2 Kazakh Research Institute of Oncology and Radiology, Almaty 050022, Kazakhstan; khus.um@mail.ru; 3 Department of General Medical Practice of Semey City, NJSC “Semey Medical University”, Semey 071400, Kazakhstan; gul6868@mail.ru; 4 LLC «Next Event Group», Astana 010000, Kazakhstan

**Keywords:** fear of cancer progression, fear of recurrence, quality of life, prostate cancer, male reproductive cancers, androgen deprivation therapy, rehabilitation, Kazakhstan, psycho-oncology, cross-sectional study

## Abstract

**Highlights:**

**What are the main findings?**

- Fear of cancer progression (FoP) is common among Kazakhstani men treated for reproductive system cancers and is most strongly associated with current disease status (recurrence/recent treatment) and receipt of hormonal therapy.
- All individual effect sizes were small (Cohen’s d < 0.3), indicating that no single demographic or clinical factor identifies fear of progression on its own.

**What are the implications of the main findings?**

- Psychosocial support should remain available to all patients, while proactive screening may be particularly useful for those under active treatment/with recurrent disease and those receiving hormonal therapy, using data already available in routine records.
- A validated Russian/Kazakh-language fear-of-progression instrument is needed before these findings can inform individual-level clinical decisions.

**Abstract:**

**Background:** Fear of cancer progression (FoP) is a well-recognised, clinically significant dimension of quality of life in oncology, yet it remains largely unstudied among men treated for reproductive system malignancies in Central Asia. This study assessed the level and correlates of FoP among such patients across several regions of Kazakhstan, as part of a broader research programme aimed at improving rehabilitation services for this patient group. **Methods:** A cross-sectional survey was conducted among 1081 men treated for a malignancy of the reproductive system, recruited across seven administrative regions of Kazakhstan (Pavlodar, Karaganda, Astana, Shymkent, Kyzylorda, Taldykorgan and East Kazakhstan). Participants completed a 12-item, 5-point Likert-scale questionnaire covering the same thematic domains as the Fear of Progression Questionnaire–Short Form (FoP-Q-SF), together with a structured sociodemographic and clinical questionnaire. Total FoP scores were calculated, together with two post hoc, exploratory subscales (somatic/health-related and social/occupational-family), reported descriptively only. Associations with sociodemographic and clinical variables were examined using *t*-tests, one-way ANOVA with Tukey post hoc comparisons, Spearman correlation and multivariable linear regression. **Results:** Internal consistency of the 12-item instrument was good (Cronbach’s α = 0.864). The mean total FoP score was 38.6 ± 8.2 (observed and possible range, 12–60). Patients who had experienced disease recurrence or had an unclear disease status scored markedly higher than patients with no recurrence and no treatment in the preceding six months (mean difference 4.07 points, *p* < 0.001), as did patients who had received treatment in the past six months without recurrence (mean difference 2.90 points, *p* < 0.001). Patients receiving hormonal (androgen-deprivation-type) therapy reported higher fear scores than those who were not (39.4 vs. 37.7, *p* = 0.001), an association that remained significant after multivariable adjustment (β = 1.19, *p* = 0.024). Urban residents scored higher than rural residents (38.9 vs. 37.6, *p* = 0.019). Age, having minor children, and ethnicity were not significantly associated with FoP. In multivariable analysis, disease status and hormonal therapy were the only independent predictors of FoP (model R^2^ = 0.053, *p* < 0.001). **Conclusions:** Fear of cancer progression is common and clinically relevant among Kazakhstani men treated for reproductive system cancers, and was most strongly associated with current disease status and receipt of hormonal therapy rather than with age or family circumstances. These findings support integrating structured psychological screening into routine rehabilitation pathways for this patient group, with universal access to psychosocial support and proactive outreach particularly considered for patients with recurrent or recently treated disease and those receiving hormonal therapy.

## 1. Introduction

Cancer of the male reproductive system, chiefly prostate and testicular cancer, represents a substantial and growing share of the global cancer burden: prostate cancer alone accounted for an estimated 1.4 million new cases worldwide in 2020, making it one of the most common cancers in men [1]. Testicular cancer, though far less common, has also shown a rising global incidence, particularly among younger men; the two malignancies differ substantially in typical age at diagnosis, natural history and prognosis, and are considered jointly in the present study only for pragmatic sampling reasons, not because they are assumed to be clinically equivalent. In Kazakhstan, national cancer-registry analyses have documented a substantial and heterogeneous rise in prostate cancer incidence over the past two decades, with pronounced variation across the country’s provinces and a marked screening-related fluctuation between 2013 and 2017 [2,3,4]. As earlier detection and modern combinations of surgery, radiotherapy, chemotherapy and hormonal therapy extend survival, growing attention has shifted from disease control alone toward the psychological and functional consequences of treatment [5,6]. Interview-based studies across oncology settings estimate that some combination of depression, anxiety or adjustment disorder affects 30–40% of cancer patients, underscoring that psychological morbidity is not a marginal concern but a core dimension of cancer care [7].

Among these consequences, fear of cancer progression (FoP)—also referred to in the literature as fear of cancer recurrence (FCR)—has emerged as one of the most frequently reported and most persistent unmet needs of cancer survivors [8]. FoP is defined as a patient’s fear, worry or concern that the cancer might come back or progress or that current treatment might fail, and it extends beyond straightforward anxiety to encompass concerns about family, occupation, and loss of autonomy [5]. An influential cognitive-behavioural formulation conceptualises FoP as arising from the interaction of internal bodily cues and external triggers with a patient’s illness representations and coping resources, producing both affective distress and behavioural responses such as excessive symptom checking [6]. A systematic review of quantitative studies confirms that clinically significant FoP affects a substantial minority of survivors across cancer types and tends to persist over time rather than resolving spontaneously [8]. Longitudinal work in prostate cancer survivors has shown that FoP can remain elevated for many years after treatment and is directly linked to reduced health-related quality of life (QoL) [9]. In postoperative prostate cancer cohorts, FoP has been shown to affect global health status both directly and indirectly, through the coping strategies patients adopt [10].

Androgen-deprivation-type hormonal therapy, one of the mainstays of treatment specifically for prostate cancer (rather than of reproductive system malignancies as a class), carries its own well-documented psychological burden. Population-based cohort studies have found that hormonal therapy is associated with a significantly increased risk of clinically diagnosed anxiety, particularly with longer treatment duration [11], and studies conducted specifically at the start of cancer rehabilitation have shown that prostate cancer-related anxiety is already prominent at this stage of the care pathway [12]. A recent multicentre analysis of over 4000 uro-oncological patients likewise found clinically relevant psychosocial distress to be common after radical surgery, with fear of disease progression identified as a key driver [13]. Testicular cancer, which typically affects younger men, carries a comparable psychosocial burden: systematic reviews report that FoP affects roughly one-third of testicular cancer survivors and is associated with greater overall psychological distress, independent of tumour histology or treatment modality [14]. Residential setting is another factor increasingly recognised as shaping cancer survivorship experience: rural cancer survivors in several health systems report worse self-reported health, higher psychological distress and reduced access to supportive psycho-oncological services compared with their urban counterparts [15].

Despite the size and growth of the affected population in Kazakhstan, and despite the country’s own contribution to the epidemiological literature on male reproductive cancers [2,3], no published data describe the burden or correlates of fear of cancer progression among Kazakhstani men treated for these malignancies. This gap is particularly relevant for health-system planning, since FoP is a modifiable target for structured psycho-oncological intervention and pelvic-floor/psychosocial rehabilitation programmes, rather than an inevitable consequence of cancer treatment [5,8]. Impairment-driven models of cancer rehabilitation emphasise that psychological impairments should be screened and addressed across the entire care continuum, alongside physical impairments, in order to maximise survivors’ quality of life [16]; ongoing reforms to Kazakhstan’s rehabilitation system, including the 2024 expansion of state-funded rehabilitation services to patients with malignant neoplasms, make this an especially timely question for national health policy [17].

This study was conducted as part of a broader research programme aimed at improving rehabilitation measures for men treated for reproductive system oncological disease. The specific objective of the present analysis was to describe the level of fear of cancer progression among a multi-regional sample of Kazakhstani men treated for such malignancies, and to identify the sociodemographic and clinical characteristics independently associated with higher FoP, in order to inform the design of targeted psychosocial components of rehabilitation care.

## 2. Materials and Methods

### 2.1. Study Design and Setting

This was a cross-sectional, questionnaire-based study conducted across seven administrative regions of the Republic of Kazakhstan: Pavlodar, Karaganda, Astana, Shymkent, Kyzylorda, Taldykorgan and East Kazakhstan regions. Data collection formed part of a larger institutional study on the organisation and effectiveness of rehabilitation measures for men with oncological diseases of the reproductive system, coordinated by NAO “Medical University of Semey”. Data were collected between February and December 2024. The study was conducted in accordance with the principles of the Declaration of Helsinki and was approved by the Ethics Committee of Semey Medical University (Protocol No. 2, dated 12 December 2023).

### 2.2. Participants

**Figure 1:**
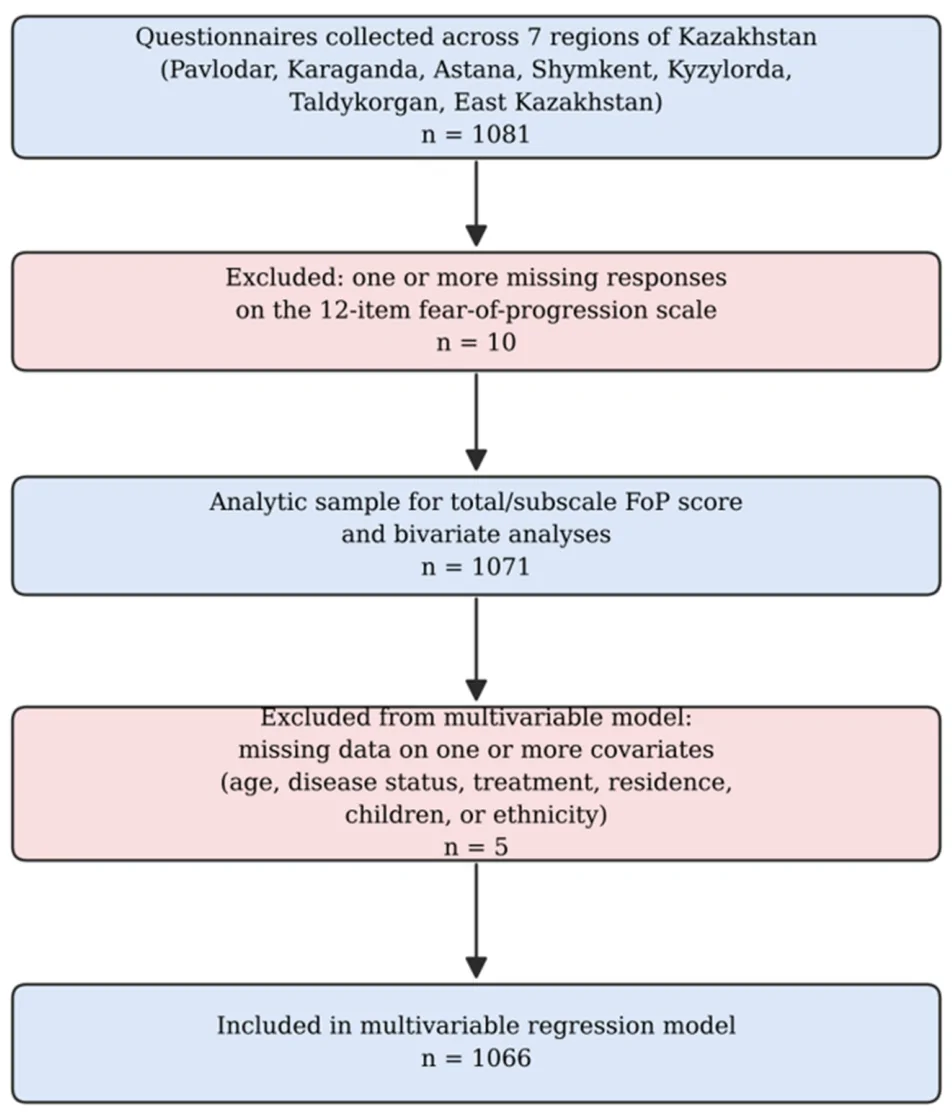
Participant flow through data cleaning and analysis stages.

Eligible participants were adult men who had provided voluntary informed consent, were resident in one of the seven study regions, and had a documented history of specialised treatment for an oncological disease of the reproductive system. Participants were surveyed regardless of current disease status, allowing the sample to include men with no evidence of recurrence, men currently undergoing surveillance or treatment, and a smaller group with recurrent or clinically unclear disease. Recruitment followed a consecutive, facility-based sampling approach within each participating region rather than a pre-specified target sample size; consequently, no formal a priori power calculation was performed, and the realised sample size (n = 1081) was determined by the number of eligible patients accessible through participating oncology services during the study period. Given the achieved sample size, the study had adequate precision for the primary comparisons reported below (disease status, hormonal therapy, residence); precision for the smaller regional subgroups (e.g., Shymkent, n = 17) is correspondingly more limited, as noted in Section 4.8. A total of 1081 questionnaires were included in the analysis (Figure 1).

### 2.3. Instrument

This article reports a focused analysis of the fear-of-cancer-progression (FoP) component of a larger, multi-domain survey administered within the parent rehabilitation study. FoP was assessed using a 12-item interviewer-administered questionnaire addressing fear of cancer progression and related psychosocial concerns, scored on a 5-point agreement scale (1 = completely disagree, 5 = completely agree); trained study personnel read the items to participants and recorded responses, which is consistent with the medico-sociological survey design of the parent study and the largely elderly composition of the sample. For completeness, we note that the original study protocol had specified the EORTC QLQ-C30 (Quality of Life Questionnaire, version 3.0), a 30-item instrument comprising five functional scales, three symptom scales, a global health status scale and additional single items [18], as the instrument for assessing broader health-related quality of life; the QLQ-C30 was not the instrument retained in the analytic dataset used for this article, and no QLQ-C30 functional, symptom, or global health status scores are reported here. Fear of progression is a well-established and clinically meaningful target of assessment in its own right, and instruments of this type have themselves previously been validated and published in *Healthcare* [19,20], supporting the suitability of reporting this component as a focused, self-contained analysis.

The 12-item questionnaire was scored on a 5-point agreement scale (1 = completely disagree, 5 = completely agree). Its item content was constructed to cover the same four thematic domains addressed by the widely used Fear of Progression Questionnaire–Short Form (FoP-Q-SF: affective/health-related reactions, family and partnership concerns, occupation, and loss of autonomy) [5], and the item wording, response format and total score range (12–60, higher scores indicating greater fear) parallel those of the FoP-Q-SF and its closely related FoP-Q-12 variant as used in oncology and cancer-rehabilitation populations internationally [5,10,21]. It should be noted that this instrument has not itself undergone formal cross-cultural psychometric validation in a Russian/Kazakh-speaking population, in contrast with the FoP-Q-SF’s existing validations in German, Portuguese, Turkish, Korean and other languages [5,19]; this is addressed as a limitation below. The questionnaire was administered to participants in Russian; the item wording reported in Section 3.2 reflects the study team’s working English translation prepared for reporting purposes, and this translation has not undergone a formal forward–back-translation or expert-panel face-validation procedure. Consequently, the precise semantic equivalence of individual items to the original FoP-Q-SF wording cannot be assured, and this is a further reason for caution in comparing item-level results with those from validated non-Russian versions of the instrument.

For descriptive purposes, two content-derived subscales were computed post hoc: a somatic/health-anxiety subscale (six items addressing fear of progression, nervousness before medical visits, fear of pain, physical anxiety symptoms, fear of burdensome procedures, and worry about medication) and a social/occupational-family subscale (six items addressing work productivity, fear that children may develop cancer, dependence on others, loss of valued activities, worry about family, and fear of being unable to work). This grouping was based on item content and mirrors, at a descriptive level, the affective/social-family structure reported for the original instrument [5]; it was not derived from a formal factor analysis of the present dataset.

The same questionnaire collected sociodemographic information (age, urban/rural residence, ethnicity, occupation, presence of children under 17 years) and clinical information (disease status—no recurrence with no treatment in the past six months, no recurrence but treatment in the past six months, or recurrence/clinically unclear status; number of tumours; and receipt of surgery, radiotherapy, chemotherapy and hormonal therapy).

### 2.4. Statistical Analysis

Descriptive statistics (mean ± standard deviation for continuous variables, frequency and percentage for categorical variables) were used to characterise the sample. Internal consistency of the total scale and of each subscale was assessed with Cronbach’s α [22]. Bivariate associations between total FoP score and categorical variables were assessed with independent-samples *t*-tests (two-level variables) or one-way ANOVA with Tukey’s honestly significant difference (HSD) post hoc test (multi-level variables); the association with age was assessed with Spearman’s rank correlation. Independent predictors of total FoP score were identified using multivariable linear regression, adjusting simultaneously for age, disease status, receipt of hormonal therapy, chemotherapy, radiotherapy and surgery, urban/rural residence, presence of minor children, and ethnicity. Multicollinearity among regression predictors was assessed using variance inflation factors (VIFs). Statistical analyses were performed using IBM SPSS Statistics, version 23.0 (IBM Corp., Armonk, NY, USA), and Python, version 3.12, with the pandas 2.x, SciPy 1.x, and statsmodels 0.14 libraries.

## 3. Results

### 3.1. Sample Characteristics

The final analytic sample comprised 1081 men, recruited across seven regions in the following proportions: East Kazakhstan 57.4% (n = 620), Pavlodar 20.7% (n = 224), Karaganda 6.8% (n = 73), Astana 5.3% (n = 57), Kyzylorda 5.1% (n = 55), Taldykorgan 3.2% (n = 35), and Shymkent 1.6% (n = 17); this marked imbalance reflects the recruitment structure of the parent study and is addressed in Section 4.8; the regional imbalance should be kept in mind when interpreting the region-specific comparisons reported in Section 3.4, particularly for the smaller regions. Mean age was 70.5 ± 9.5 years (range 25–94), and the large majority of participants (90.3%) were aged 60 years or older; 84.8% were retired. Most participants were urban residents (76.9%) and self-identified as Russian (62.8%) or Kazakh (30.2%) ethnicity. Regarding disease status, 48.9% reported no recurrence and no treatment within the previous six months, 43.7% reported no recurrence but ongoing or recent treatment, and 7.3% reported recurrence or a clinically unclear status. A majority had received surgical treatment (58.5%) and/or hormonal therapy (53.2%); 35.6% had received radiotherapy and 27.3% chemotherapy. Full sample characteristics are shown in Table 1.

### 3.2. Level of Fear of Cancer Progression

Internal consistency of the 12-item scale was good (Cronbach’s α = 0.864); for the two post hoc, exploratory subscales, internal consistency was acceptable (α = 0.777 for both). The mean total FoP score was 38.6 ± 8.2 (median 38; interquartile range 33–44; possible and observed range 12–60). For descriptive purposes, the mean scores were 19.7 ± 4.4 for the somatic/health-related subscale and 18.9 ± 4.5 for the social/occupational-family subscale (each on a 6–30 range).

At the item level (Table 2), the highest mean scores were observed for fear of pain, fear of disease progression itself, and worry about what would happen to the family, while the lowest mean score was observed for fear of depending on strangers in daily life.

**Figure 2:**
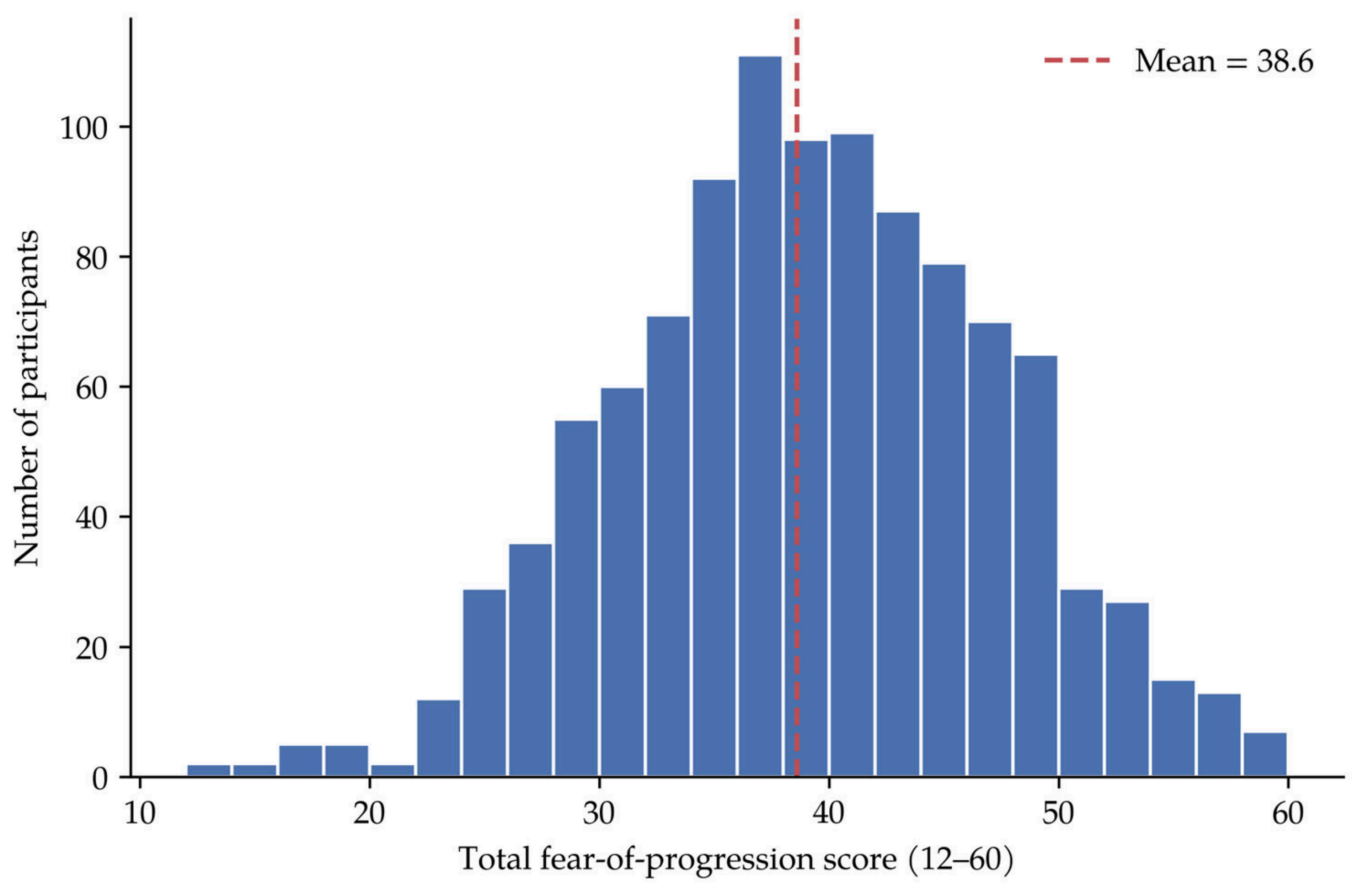
Distribution of total fear-of-progression scores across the analytic sample (N = 1071).

Figure 2 shows the distribution of total scores across the analytic sample.

### 3.3. Psychometric Properties

To evaluate the internal structure of the 12-item instrument beyond the overall Cronbach’s α reported above, corrected item-total correlations and the change in α if each item were deleted were computed (Table 3). All items correlated moderately to strongly with the remaining scale (corrected item-total r = 0.45–0.66), and removal of no single item would have improved overall reliability (all α-if-deleted values were at or below the observed overall α of 0.864), supporting retention of all 12 items.

An exploratory factor analysis (principal-axis extraction, varimax rotation) was conducted to examine the dimensional structure of the 12 items and to check the a priori somatic/social-family grouping used descriptively above. Sampling adequacy was excellent (Kaiser–Meyer–Olkin measure = 0.89) and Bartlett’s test of sphericity was significant (χ^2^ = 4344.7, df = 66, *p* < 0.001), confirming the correlation matrix was suitable for factor analysis. A two-factor solution was retained on the basis of the scree pattern (eigenvalues 4.87 and 1.34 for the first two factors, dropping to 0.95 for the third), together accounting for 51.7% of total variance (Table 4). The first, dominant factor was defined primarily by items concerning family, work, dependency, and burdensome procedures, while the second, smaller factor was defined primarily by the three core affective items (fear of progression itself, nervousness before visits, and fear of pain). This empirical structure did not fully reproduce the a priori somatic/social-family split used for the descriptive subscales in Section 3.2: several items originally assigned to the somatic subscale (e.g., fear of burdensome procedures, worry that medication may harm the body) loaded most strongly on the broader first factor alongside the social/occupational items. Given the size of the first eigenvalue relative to the second and the item cross-loadings observed, these results are most consistent with a single dominant general factor accompanied by a smaller, more specific affective-symptom factor, rather than two clearly separable and equally weighted subscales. Accordingly, the total score—which was the primary outcome in all inferential analyses reported below—is better supported by these data than the two descriptive subscales, which should be interpreted cautiously and regarded as provisional pending formal confirmatory validation.

Taken together, these psychometric findings do not establish this instrument as equivalent to the validated FoP-Q-SF, and it should not be presented or used as such. Although the questionnaire was developed to parallel the conceptual structure of the FoP-Q-SF rather than to replace it, the internal consistency, item-total correlation, and exploratory factor-analytic findings reported above suggest acceptable reliability and a coherent, interpretable internal structure adequate for exploratory epidemiological research of the kind reported here. Nevertheless, formal linguistic (forward–back-translation) and psychometric (confirmatory factor analysis, test–retest reliability, convergent validity) validation of a Russian- and Kazakh-language fear-of-progression instrument remains necessary before any clinical implementation, individual-level scoring, or cross-study comparison of item-level results.

### 3.4. Bivariate Associations

**Figure 3:**
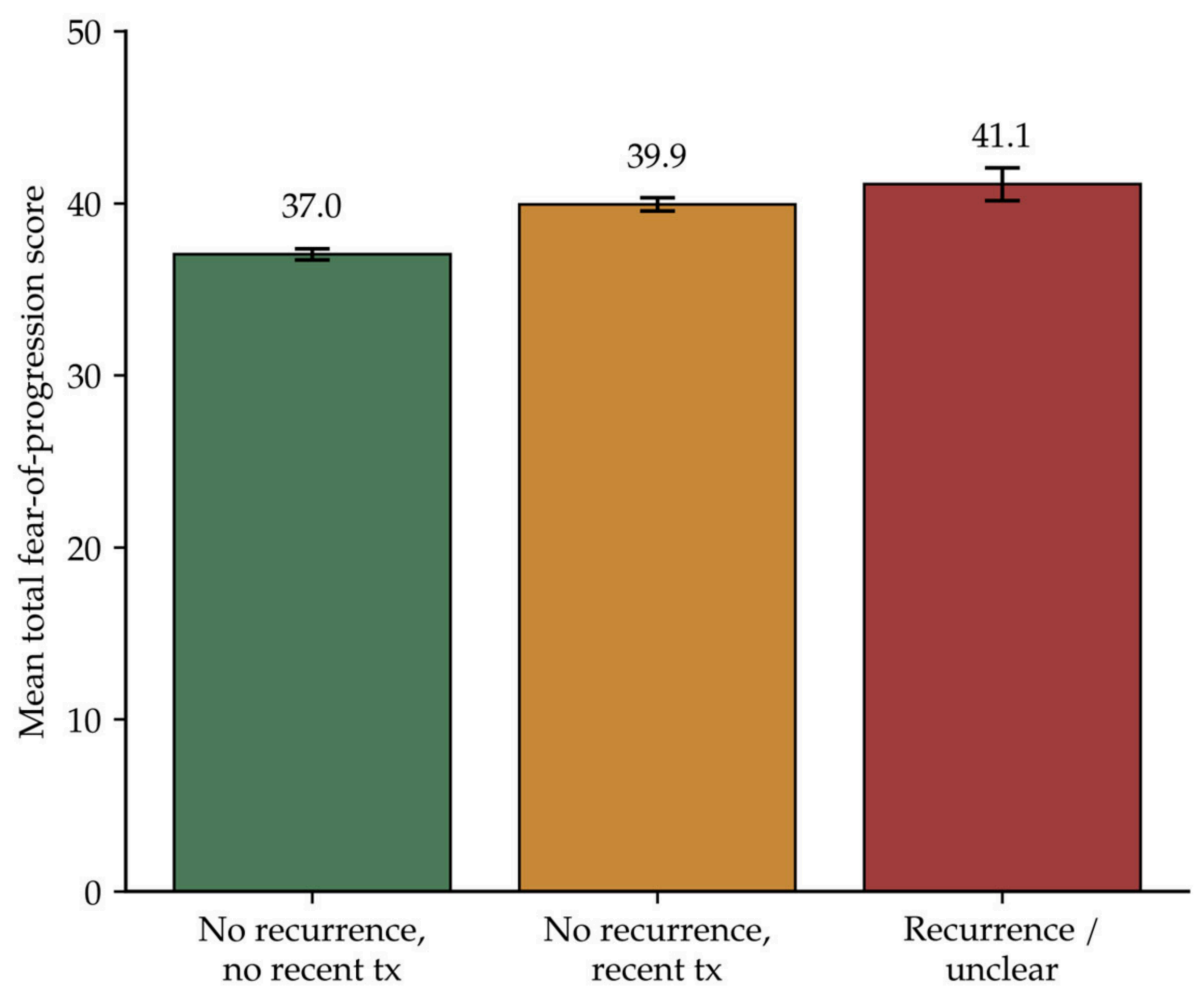
Mean total fear-of-progression score by disease status. Error bars represent the standard error of the mean; one-way ANOVA F = 20.38, *p* < 0.001 (see Table 5 and Section 3.4).

Total FoP score differed significantly by current disease status (one-way ANOVA, F = 20.38, *p* < 0.001, partial η^2^ = 0.037, a small-to-moderate effect). Post hoc comparisons showed that patients with no recurrence and no recent treatment scored significantly lower than both patients with no recurrence but recent treatment (mean difference 2.90 points, *p* < 0.001) and patients with recurrence or unclear status (mean difference 4.07 points, *p* < 0.001); the difference between the latter two groups was not statistically significant (*p* = 0.458). Figure 3 illustrates this pattern.

Region of residence was also significantly associated with FoP score (F = 10.77, *p* < 0.001, partial η^2^ = 0.057): participants from Shymkent (mean 49.1, n = 17) and Astana (mean 43.8, n = 57) reported markedly higher scores than participants from the remaining regions (means 36.8–40.2), which did not differ substantially from one another. Urban residents scored higher than rural residents (38.9 vs. 37.6, t = 2.36, *p* = 0.019, Cohen’s d = 0.17, a small effect). Participants receiving hormonal therapy scored higher than those not receiving it (39.4 vs. 37.7, t = 3.49, *p* = 0.001, d = 0.21); a similar, borderline pattern was observed for chemotherapy (39.4 vs. 38.3, *p* = 0.052, d = 0.14), while radiotherapy (*p* = 0.376, d = −0.06) and surgery were not significantly associated with FoP score. Age showed no meaningful correlation with FoP score (Spearman ρ = −0.0004, *p* = 0.988), and presence of minor children was not significantly associated with FoP (*p* = 0.525, d = 0.07). All effect sizes for the significant bivariate associations were small in conventional terms (d < 0.3; partial η^2^ < 0.06), a point taken up in Section 4.7. Table 5 summarises these bivariate comparisons.

### 3.5. Multivariable Analysis

**Figure 4:**
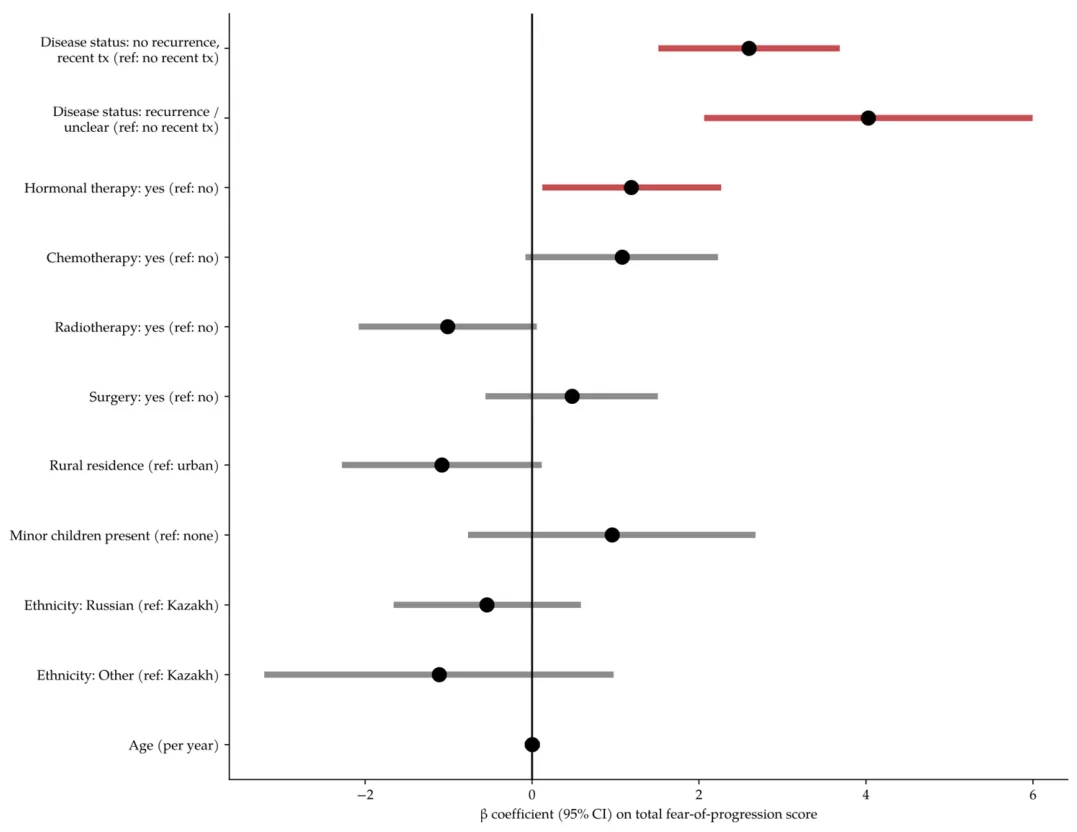
Forest plot of multivariable regression coefficients (β, 95% CI) for total fear-of-progression score. Red intervals do not cross zero.

In a multivariable linear regression model adjusting simultaneously for age, disease status, hormonal therapy, chemotherapy, radiotherapy, surgery, urban/rural residence, presence of minor children and ethnicity (n = 1066, R^2^ = 0.053, adjusted R^2^ = 0.044, F = 5.41, *p* < 0.001), disease status remained the strongest independent predictor of FoP score, and hormonal therapy remained independently associated with higher FoP after adjustment. Radiotherapy and rural residence showed borderline independent associations with lower FoP score. Age, surgery, chemotherapy, presence of minor children and ethnicity were not independent predictors once other variables were accounted for (Table 6, Figure 4). Standardised coefficients confirmed disease status as the dominant predictor (standardised β = 0.32 and 0.49 for the two disease-status contrasts, versus β = 0.15 for hormonal therapy and β ≤ 0.13 for all other significant or borderline predictors), indicating that, among the variables measured, how recently a patient had been treated or whether disease had recurred mattered more to fear of progression than any other factor examined.

Variance inflation factors for all predictors ranged from 1.04 to 1.20, indicating no meaningful multicollinearity among the treatment-modality variables despite their expected co-occurrence in combined treatment regimens (Supplementary Table S1). Residual diagnostics indicated a statistically significant but numerically minor departure from normality (Shapiro–Wilk W = 0.997, *p* = 0.027) and borderline evidence of heteroscedasticity (Breusch–Pagan *p* = 0.052); both results are unsurprising given the large sample size, which affords high power to detect small deviations from these idealised assumptions, and neither is of a magnitude expected to materially bias the coefficient estimates. No individual observation showed a Cook’s distance exceeding 1 (maximum observed = 0.019), indicating that the results were not driven by a small number of unduly influential cases. Visual inspection of the residuals-versus-fitted and normal Q–Q plots did not suggest substantial departures from the model’s linearity or normality assumptions (Supplementary Figure S1).

### 3.6. Additional Exploratory Analyses

Two pre-specified interaction terms were tested to probe whether the effects identified above varied across subgroups. The interaction between hormonal therapy and disease status was not statistically significant for the no-recurrence/recent-treatment contrast (*p* = 0.504) but showed a borderline trend for the recurrence/unclear contrast (*p* = 0.078), consistent with a possible ceiling effect in which the impact of hormonal therapy on fear is attenuated among patients already experiencing the highest fear levels (recurrent disease); given the modest cell size for this subgroup (n = 78 with recurrence, further split by hormonal therapy status), this finding should be regarded as hypothesis-generating rather than confirmatory. The interaction between urban/rural residence and age was not statistically significant (*p* = 0.103), indicating that the urban–rural difference in FoP did not vary appreciably by age. Both interaction terms were dropped from the final multivariable model (Table 6) in favour of the simpler main-effects specification, which is preferred both for parsimony and because neither interaction reached conventional significance.

An exploratory cumulative treatment-count variable (sum of surgery, radiotherapy, chemotherapy and hormonal therapy received, range 0–4) showed a weak but statistically significant positive association with FoP score (Spearman ρ = 0.063, *p* = 0.041), and mean FoP scores increased in a broadly monotonic, if modest, pattern from patients who had received no listed treatment (37.5 ± 8.3, n = 120) to those who had received all four (40.3 ± 10.0, n = 70), though the corresponding one-way ANOVA fell just short of conventional significance (F = 2.18, *p* = 0.070). This pattern is consistent with, but does not on its own establish, a cumulative treatment-burden contribution to fear of progression that is at least partly captured by, but not fully reducible to, the individual treatment-modality effects reported above.

## 4. Discussion

### 4.1. Level of Fear of Cancer Progression in Context

This is, to our knowledge, the first multi-regional description of fear of cancer progression among men treated for reproductive system malignancies in Kazakhstan. Three findings stand out. First, the overall level of FoP in this sample (mean 38.6 ± 8.2 on a 12–60 scale) was substantial and, notably, somewhat higher than that reported in a comparable postoperative prostate cancer cohort in China using the same 12-item, 5-point format (34.3 ± 5.9) [10]. This difference may partly reflect the composition of our sample, which—unlike the Chinese cohort of exclusively post-surgical patients—also included men still undergoing treatment and a smaller group with recurrent or unclear disease, both of which were associated with higher fear in our own data.

A further point that bears directly on the interpretation of these findings, and not only on their limitations, is that prostate and testicular cancer are clinically distinct diseases: they differ markedly in typical age at diagnosis, natural history, treatment pathway and prognosis, and the psychosocial support strategies appropriate to each are correspondingly different. The present dataset did not distinguish between the two diagnoses, and the two groups are combined here as “men treated for reproductive system malignancies” purely because clinical records did not allow finer stratification. Given that the sample skewed strongly toward older, retired men (mean age 70.5 years), the results reported below almost certainly reflect predominantly, though not exclusively, the experience of men treated for prostate cancer; findings should not be assumed to generalise directly to younger testicular cancer survivors, whose disease trajectory, life stage and support needs differ substantially. We return to this point, and its implications for future data collection, in Section 4.8.

### 4.2. Disease Status as the Principal Correlate

Second, and consistent with the broader oncology literature, current disease status was by far the strongest correlate of FoP: patients with no evidence of recurrence and no recent treatment reported the lowest fear, while those with ongoing treatment or recurrence reported substantially higher fear, a pattern that persisted after multivariable adjustment. This mirrors findings from longitudinal prostate cancer cohorts showing that FoP tracks closely with objective indicators of disease activity and treatment burden rather than diminishing steadily with time since diagnosis [9]. The lack of a significant difference between patients on recent treatment and those with recurrence suggests that, from the patient’s perspective, active management and confirmed recurrence may carry a similarly heavy psychological load—a distinction that may be less salient to patients than it is to clinicians.

### 4.3. Hormonal Therapy and Psychological Burden

Third, receipt of hormonal therapy was independently associated with higher FoP even after adjusting for disease status. This finding is consistent with a substantial body of evidence linking androgen-deprivation-type therapy to elevated anxiety in men with prostate cancer, including population-based cohort data showing an increased hazard of clinically diagnosed anxiety with hormonal therapy exposure, particularly with longer treatment duration [11]. Androgen deprivation is thought to act on mood and anxiety through both a direct neuroendocrine pathway and the burden of its physical side effects (hot flashes, sexual dysfunction, fatigue), any of which could plausibly heighten a patient’s day-to-day awareness of illness and, in turn, fear of progression. This is consistent with earlier observations that prostate cancer-related anxiety is already prominent at the start of cancer rehabilitation [12], and with more recent multicentre evidence that fear of progression is a key driver of post-surgical psychosocial distress in uro-oncological patients [13]. This finding suggests that hormonal therapy pathways may represent an appropriate setting for proactive psychological screening, rather than screening being offered only at diagnosis or at the point of surgery.

### 4.4. Urban–Rural Differences

The association between urban residence and higher FoP was smaller in magnitude but notable because it runs counter to much of the rural–urban disparities literature from other health systems, where rural cancer survivors more often report worse psychological outcomes, attributed to reduced access to supportive and psycho-oncological services [15]. In the Kazakhstani context, several explanations are plausible and cannot be distinguished with the present cross-sectional design: urban patients may have greater exposure to information (including online information) about prognosis and recurrence that heightens awareness and worry; urban residence may correlate with more intensive surveillance and more frequent contact with oncology services, each visit constituting a reminder of illness; or rural patients may under-report psychological symptoms because of cultural or generational factors. This finding merits targeted qualitative follow-up rather than causal interpretation.

### 4.5. Age and Generalisability

The absence of an association between age and FoP is noteworthy given that some (though not all) studies in the international literature report higher FoP among younger patients, often attributed to greater disruption of work, family and life plans [5]. In the present sample—overwhelmingly composed of retired men aged 60 years and above, with fewer than 3% under 50—the restricted age range and near-universal retirement status may have attenuated any age-related gradient that might be more visible in a younger, working-age cohort. This should be considered when generalising these findings to younger patients with reproductive system malignancies, including testicular cancer, which more typically affects men earlier in adulthood and for which fear of recurrence has been shown to follow a somewhat different course, with roughly one-third of survivors affected regardless of treatment modality [14]. A related interpretive issue concerns the two occupation-referenced items (fear of reduced work productivity; fear of being unable to work): with 84.8% of the sample retired, it is unclear how these items were understood by respondents no longer in employment—whether as a hypothetical concern, a recollection of earlier working life, or as not applicable. The absence of a meaningful age gradient in overall FoP scores suggests this did not materially distort the total score, but item-level responses to these two questions specifically should be interpreted with caution. Future administrations of this instrument in largely retired populations should consider either rewording these items or analysing them separately by employment status.

### 4.6. Implications for Rehabilitation Practice

Taken together, these findings have direct relevance to the design of rehabilitation programmes for this patient group. Impairment-driven models of cancer rehabilitation argue that psychological impairments should be screened and treated with the same systematic rigour as physical ones across the entire care continuum [16]; the present results suggest a practical way to operationalise this principle locally. Every patient in this population may benefit from psychosocial support, and access to it should not be restricted to any subgroup. Within that principle of universal access, however, services with limited capacity may still choose to reach out proactively to two readily identifiable subgroups likely to be at higher risk: patients currently undergoing treatment or with recurrent/unclear disease status, and patients receiving hormonal therapy—both of which can be flagged from information already routinely available in clinical records. Such proactive outreach is intended to complement, not substitute for, open access to psychological support for every patient who wants it. Kazakhstan’s recent extension of state-funded rehabilitation services to patients with malignant neoplasms, and its experience developing mobile and home-based supportive care models for oncology patients, provide existing infrastructure into which such targeted psychosocial screening could realistically be integrated [17,23].

#### Clinical Implications for Psycho-Oncology in Kazakhstan

Psycho-oncology as a distinct clinical and research discipline is still developing in Kazakhstan and, more broadly, across Central Asia, where oncology services have historically prioritised surgical and pharmacological treatment capacity over structured psychosocial support. The present findings offer a concrete, low-cost starting point for building this capacity. The two strongest correlates of fear of progression identified here—disease status and receipt of hormonal therapy—are already recorded in routine oncology and rehabilitation records, so services may not need a new screening infrastructure to identify groups for proactive outreach; these routinely recorded variables can help services prioritise who to approach first, while direct psychological screening remains necessary to identify individual support needs. Embedding a brief distress or fear-of-progression check into existing rehabilitation consultations, prioritised for these two groups, would represent a feasible first step toward a national psycho-oncology service model, consistent with the direction of Kazakhstan’s current rehabilitation reforms [17,23], and would generate the kind of longitudinal, intervention-linked data that this cross-sectional study cannot provide on its own. Given the increasing survivorship of men treated for prostate and other reproductive system cancers worldwide, these findings may also be relevant beyond Kazakhstan, particularly for other middle-income countries where psycho-oncology services remain similarly underdeveloped relative to the size of the affected population.

### 4.7. On Explained Variance and Clinical Significance

The multivariable model explained only a modest share of the variance in FoP scores (R^2^ = 0.053), and all individual effect sizes were small by conventional benchmarks (Cohen’s d < 0.3; partial η^2^ < 0.06). This is not, in our view, evidence against the clinical relevance of the findings, for three reasons. First, FoP is a well-established multicomponent construct: theoretical models locate its origins in the interaction of illness perceptions, coping style, attachment security, trait anxiety, and broader psychological vulnerability [6], none of which were measured in the present sociodemographic and clinical questionnaire. A model built solely from administrative and demographic variables would not be expected to explain more than a modest fraction of variance in a psychological outcome shaped so heavily by unmeasured personality and cognitive factors; the R^2^ observed here is, in that light, broadly consistent with what would be expected from a purely clinical/demographic predictor set. Second, the practical value of the disease-status and hormonal-therapy findings does not depend on the overall model R^2^: these two variables are readily available in routine clinical records and cost nothing additional to collect. They identify subgroups (recently treated or recurrent patients; patients on hormonal therapy) whose mean FoP scores differ by 2.6–4.0 points on a 48-point range—a difference of a similar order of magnitude to the between-group differences that have been judged clinically meaningful in other FoP-Q-SF-based studies [10]. Third, a small explained-variance statistic is itself informative for service design: it indicates that a single risk-stratification rule based on demographic and treatment variables alone will misclassify many individual patients. This argues for pairing routine-data screening with a brief, direct self-report check, rather than relying on record-based prediction alone—very short instruments of this kind, down to four items, have themselves been validated as adequate stand-ins for longer fear-of-progression measures in other settings [20].

### 4.8. Strengths and Limitations

Several limitations should be considered. First, the study protocol had originally specified the EORTC QLQ-C30 for quality-of-life assessment [18]; the instrument actually fielded and analysed here—a 12-item fear-of-progression questionnaire—covers only the psychosocial/fear dimension of quality of life and not the physical, functional and symptom domains captured by the QLQ-C30. Findings should therefore be interpreted as describing fear of cancer progression specifically, not overall health-related quality of life, and future data collection within this research programme should incorporate the QLQ-C30 (or confirm and report its results separately) to allow the broader quality-of-life picture described in the protocol to be addressed. Second, the fear-of-progression instrument used here, while constructed to mirror the content domains of the validated FoP-Q-SF [5,19,20,21], has not itself undergone formal psychometric validation in a Russian/Kazakh-speaking population. Internal consistency was good (α = 0.864, with all item-total correlations ≥ 0.45). An exploratory factor analysis yielded excellent sampling adequacy (KMO = 0.89) and an interpretable two-factor solution (Section 3.3); however, this exploratory structure only partly reproduced the a priori somatic/social-family subscales used descriptively in Section 3.2, and the instrument still lacks test–retest reliability data, convergent validity against an independent anxiety or quality-of-life measure, and confirmatory (as opposed to exploratory) factor-analytic support. The two content-derived subscales should therefore continue to be treated as provisional, and the total score—the primary outcome throughout—rests on firmer psychometric ground than the subscales. Third, the study did not record the specific cancer diagnosis (e.g., prostate versus testicular cancer), disease stage at diagnosis, or time since diagnosis/treatment completion, all of which are known correlates of FoP in the international literature and which future work in this cohort should incorporate. Fourth, the cross-sectional design precludes causal inference, particularly regarding the direction of the residence and hormonal-therapy associations. Relatedly, all responses were self-reported and collected via interviewer administration rather than anonymous self-completion, so they may have been affected by recall bias or social desirability bias, potentially including a tendency to under- or over-report fear in the presence of study personnel. We cannot rule out that this interviewer-administered format elicited systematically different reporting of psychological symptoms than an anonymous self-administered survey would have, and this possibility should be tested directly in future work using a self-completion arm. Fifth, participants were recruited through oncology services in each region rather than through a formal probability sampling frame, so the sample may not be fully representative of all men treated for reproductive system cancers in these regions, and the marked imbalance in regional sample sizes (from n = 17 in Shymkent to n = 620 in East Kazakhstan) limits the precision of region-specific estimates. Finally, occupational status was recorded using an open, non-standardised classification, which required collapsing into broad categories for analysis.

## 5. Conclusions

Fear of cancer progression is common and clinically meaningful among Kazakhstani men treated for reproductive system malignancies, and its intensity was primarily associated with current disease status and receipt of hormonal therapy rather than with age, ethnicity, or family circumstances. These results support embedding structured psychological screening and support within rehabilitation programmes for this patient group, with universal access to psychosocial support and proactive outreach particularly considered for patients under active treatment or with recurrent/unclear disease and for those receiving hormonal therapy. Given the modest variance explained by demographic and clinical variables alone, these recommendations should be read as health-system-level priorities for allocating rehabilitation resources, not as an individual-level diagnostic or risk-stratification tool. Future work should incorporate cancer-type-specific analysis, formal cross-cultural and linguistic (forward–back-translation) validation of the fear-of-progression instrument in Russian and Kazakh, and longitudinal follow-up to clarify the direction of the associations identified here.

## Figures and Tables

**Table 1:** Sociodemographic and clinical characteristics of study participants (N = 1081).

Characteristic	n	%
Age, years—mean ± SD (range)	70.5 ± 9.5 (25–94)	
<50	30	2.8
50–59	75	6.9
60–69	336	31.1
≥70	640	59.2
Region		
East Kazakhstan	620	57.4
Pavlodar	224	20.7
Karaganda	73	6.8
Astana	57	5.3
Kyzylorda	55	5.1
Taldykorgan	35	3.2
Shymkent	17	1.6
Residence		
Urban	831	76.9
Rural	250	23.1
Ethnicity		
Kazakh	326	30.2
Russian	679	62.8
Other	74	6.8
Occupational status		
Retired/pensioner	917	84.8
Not employed	59	5.5
Employed (various occupations)	104	9.6
Minor children (<17 years)	104	9.6
Disease status		
No recurrence, no treatment in last 6 months	529	48.9
No recurrence, treatment in last 6 months	472	43.7
Recurrence/clinically unclear	79	7.3
Number of tumours (primary malignant lesions recorded per patient)		
One	867	80.2
Two	131	12.1
Unknown	80	7.4
Treatment received		
Surgery	632	58.5
Radiotherapy	385	35.6
Chemotherapy	295	27.3
Hormonal therapy	575	53.2

SD, standard deviation. Percentages are calculated on the non-missing responses for each variable (missing data ranged from 0 to 3 observations per variable).

**Table 2:** Item-level descriptive statistics for the 12-item fear-of-progression instrument, ranked by mean score (N = 1073–1081).

Item	Mean	SD
Fear of pain	3.40	1.06
Fear of disease progression	3.40	1.10
Worry about what will happen to the family	3.40	1.04
Fear of burdensome medical procedures	3.38	1.02
Fear that children may develop cancer	3.26	1.16
Worry that medication may harm the body	3.24	1.04
Fear of being unable to pursue favourite activities	3.16	1.05
Physical anxiety symptoms (palpitations, abdominal pain, nervousness)	3.15	1.02
Nervousness before medical visits/check-ups	3.11	1.13
Fear of becoming less productive at work	3.11	1.08
Fear of being unable to work	3.01	1.08
Fear of depending on strangers in daily life	2.96	1.09

Items were rated on a 5-point scale (1 = completely disagree, 5 = completely agree). SD, standard deviation.

**Table 3:** Item-total correlations and reliability if item deleted (N = 1071).

Item	Corrected Item-Total r	α If Item Deleted
Fear of disease progression	0.448	0.860
Nervousness before medical visits/check-ups	0.544	0.853
Fear of pain	0.557	0.853
Fear of becoming less productive at work	0.477	0.858
Physical anxiety symptoms	0.482	0.857
Fear that children may develop cancer	0.581	0.851
Fear of depending on strangers in daily life	0.565	0.852
Fear of being unable to pursue favourite activities	0.662	0.846
Fear of burdensome medical procedures	0.655	0.846
Worry that medication may harm the body	0.593	0.850
Worry about what will happen to the family	0.502	0.856
Fear of being unable to work	0.475	0.858

Overall scale α = 0.864 (N = 1071 complete cases).

**Table 4:** Exploratory factor analysis: Rotated factor loadings for the 12-item fear-of-progression instrument (N = 1071).

Item	Factor 1	Factor 2
Fear of disease progression	0.122	0.743
Nervousness before medical visits/check-ups	0.172	0.838
Fear of pain	0.225	0.784
Fear of becoming less productive at work	0.437	0.354
Physical anxiety symptoms	0.448	0.358
Fear that children may develop cancer	0.698	0.181
Fear of depending on strangers in daily life	0.526	0.398
Fear of being unable to pursue favourite activities	0.721	0.278
Fear of burdensome medical procedures	0.729	0.261
Worry that medication may harm the body	0.741	0.147
Worry about what will happen to the family	0.714	0.020
Fear of being unable to work	0.584	0.146
Eigenvalue	4.87	1.34
% variance explained	30.8%	20.8%

Extraction method: principal-axis factoring; rotation: varimax. Loadings ≥ 0.45 in boldface pattern are the dominant loading for each item (bolding not reproduced in plain-text table; see loading magnitudes). KMO = 0.89; Bartlett’s χ^2^(66) = 4344.7, *p* < 0.001.

**Table 5:** Total fear-of-progression score by selected sociodemographic and clinical characteristics, with effect sizes.

Characteristic	n	Mean ± SD	Test Statistic	Effect Size	*p*-Value
Disease status			F = 20.38	η^2^p = 0.037	<0.001
No recurrence, no recent tx	526	37.0 ± 7.6			
No recurrence, recent tx	466	39.9 ± 8.4			
Recurrence/unclear	78	41.1 ± 8.4			
Residence			t = 2.36	d = 0.17	0.019
Urban	822	38.9 ± 8.2			
Rural	249	37.6 ± 7.9			
Hormonal therapy			t = 3.49	d = 0.21	0.001
Yes	568	39.4 ± 8.3			
No	503	37.7 ± 7.9			
Chemotherapy			t = 1.95	d = 0.14	0.052
Yes	290	39.4 ± 8.3			
No	780	38.3 ± 8.1			
Radiotherapy			t = −0.89	d = −0.06	0.376
Yes	379	38.3 ± 8.6			
No	692	38.8 ± 7.9			
Minor children present			t = 0.64	d = 0.07	0.525
Yes	103	39.1 ± 8.2			
No	968	38.6 ± 8.1			
Age (continuous)	1071	—	ρ = −0.0004	—	0.988

SD, standard deviation; tx, treatment; d, Cohen’s d; η^2^p, partial eta-squared. Sample sizes in this table (and in the multivariable model, Table 6) are slightly lower than in Table 1 because ten participants had one or more missing responses on the 12-item fear-of-progression scale and were excluded listwise from the total-score analyses. Region-level comparisons (7 groups, F = 10.77, *p* < 0.001, η^2^p = 0.057) are described in the text rather than tabulated, owing to the number of pairwise contrasts.

**Table 6:** Multivariable linear regression predicting total fear-of-progression score (n = 1066).

Predictor	β (95% CI)	Standardised β	*p*-Value
Intercept	36.68 (32.89 to 40.46)	–	<0.001
Disease status: no recurrence, recent tx (ref: no recurrence, no recent tx)	2.60 (1.55 to 3.65)	0.32	<0.001
Disease status: recurrence/unclear (ref: no recurrence, no recent tx)	4.03 (2.10 to 5.96)	0.49	<0.001
Hormonal therapy: yes (ref: no)	1.19 (0.16 to 2.23)	0.15	0.024
Chemotherapy: yes (ref: no)	1.08 (−0.04 to 2.19)	0.13	0.058
Radiotherapy: yes (ref: no)	−1.01 (−2.04 to 0.02)	−0.12	0.054
Surgery: yes (ref: no)	0.48 (−0.52 to 1.47)	0.06	0.349
Rural residence (ref: urban)	−1.08 (−2.24 to 0.08)	−0.13	0.069
Minor children present (ref: none)	0.96 (−0.73 to 2.64)	0.12	0.265
Ethnicity: Russian (ref: Kazakh)	−0.54 (−1.62 to 0.55)	−0.07	0.335
Ethnicity: Other (ref: Kazakh)	−1.11 (−3.17 to 0.94)	−0.14	0.288
Age (per year)	0.003 (−0.05 to 0.06)	0.00	0.901

β, unstandardised regression coefficient; CI, confidence interval; tx, treatment; ref, reference category. Standardised β obtained by re-fitting the model on z-scored continuous variables and the z-scored outcome (categorical predictors retain their dummy coding). Model R^2^ = 0.053.

## Data Availability

The data are not publicly available because they contain detailed clinical and sociodemographic information collected from cancer patients. Public deposition of participant-level data was not covered by the informed consent obtained from participants or by the approval granted by the Ethics Committee of Semey Medical University (Protocol No. 2, dated 12 December 2023). Therefore, public sharing of these data would not be consistent with the participants’ consent or the conditions of the ethical approval. Access to anonymized data may be provided upon reasonable request from the corresponding author, subject to institutional and ethical approval where applicable.

## Supplementary Materials

The following supporting information can be downloaded at: https://www.mdpi.com/article/10.3390/healthcare14182961/s1, Table S1: Variance inflation factors (VIF) for predictors in the multivariable regression model (n = 1066); Figure S1: Normal Q-Q plot of model residuals (left) and standardized residuals versus leverage (right) for the multivariable regression model reported in Table 6. No observation showed both high leverage and a large standardized residual.
