# Supplementary material for: Fear of Cancer Progression as a Psychological Component of Cancer Rehabilitation in Men Treated for Reproductive System Malignancies: A Multi-Regional Cross-Sectional Study in Kazakhstan
Source: Healthcare (Basel). 2026 Sep 10;14(18):2961. doi: 10.3390/healthcare14182961 (PMC13606434; doi:10.3390/healthcare14182961)
Supplement: Supplementary file 1 [file healthcare-14-02961-s001.zip › healthcare-4460813-supplementary.pdf]

Supplementary Materials

Table S1. Variance inflation factors (VIF) for predictors in the multivariable regression model (n = 1066)

Predictor	VIF
Age	1.09
Disease status: no recurrence, treatment in the past 6 months	1.19
Disease status: recurrence / unclear	1.09
Hormonal therapy: yes	1.16
Chemotherapy: yes	1.06
Radiotherapy: yes	1.05
Surgery: yes	1.04
Rural residence	1.05
Minor children present	1.07
Ethnicity: Russian	1.20
Ethnicity: Other	1.16

VIF, variance inflation factor. All values well below the conventional concern threshold of 5, indicating no meaningful multicollinearity among model predictors.

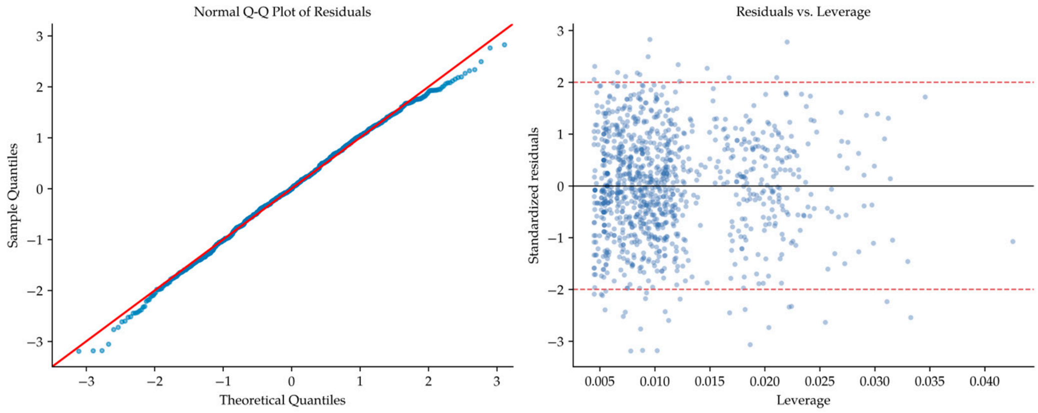

Figure S1. Normal Q-Q plot of model residuals (left) and standardized residuals versus leverage (right) for the multivariable regression model reported in Table 6. No observation showed both high leverage and a large standardized residual.
